# A New Generation of Lineage Tracing Dynamically Records Cell Fate Choices

**DOI:** 10.3390/ijms23095021

**Published:** 2022-04-30

**Authors:** Mingze Yao, Tinglin Ren, Yuanqing Pan, Xiaoqing Xue, Rong Li, Lei Zhang, Yuhang Li, Ke Huang

**Affiliations:** Institutes of Biomedical Sciences, Shanxi University, Taiyuan 030006, China; tinglinr@163.com (T.R.); pyqing0206@163.com (Y.P.); xxq13834616691@163.com (X.X.); lirong4683@163.com (R.L.); alei006@126.com (L.Z.); liyuhang1992@foxmail.com (Y.L.); hkenb@163.com (K.H.)

**Keywords:** lineage tracing, fate mapping, CRISPR/Cas9, stem cell, single-cell, gene editing

## Abstract

Reconstructing the development of lineage relationships and cell fate mapping has been a fundamental problem in biology. Using advanced molecular biology and single-cell RNA sequencing, we have profiled transcriptomes at the single-cell level and mapped cell fates during development. Recently, CRISPR/Cas9 barcode editing for large-scale lineage tracing has been used to reconstruct the pseudotime trajectory of cells and improve lineage tracing accuracy. This review presents the progress of the latest CbLT (CRISPR-based Lineage Tracing) and discusses the current limitations and potential technical pitfalls in their application and other emerging concepts.

## 1. Introduction

**Figure 1:**
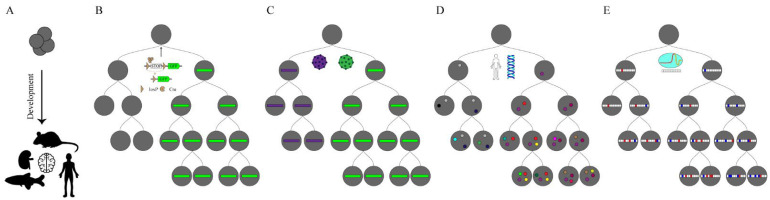
Lineage tracing in development. (**A**) The goal of lineage tracing is to capture the continuous development of a tissue, organ or individual from a fertilized egg or capture a few progenitor cells from an organism and represent them in the form of a lineage tree. (**B**) The recombinase reporter system is integrated into the cell genome and GFP (Green Fluorescent Proteins) expression is activated when the Cre (Cyclization Recombination Enzyme) enzyme is expressed in specific cells. (**C**) Based on the cellular barcoding lineage tracing, using viruses or transposons allow cells to be uniquely tagged. These markers can be inherited from offspring and are used to reconstruct the cloning relationship. However, this cannot distinguish the relationships between the progeny cells. (**D**) Somatic mutations rarely occur throughout the genome with the development of an organism (marked with colored dots). These mutations can be used to trace a complete lineage tree but require whole-genome sequencing. (**E**) CRISPR-based lineage-tracing approaches add combinatorial and cumulative information over developmental time. These approaches can reconstruct lineages between progeny and progenitor cells, effective in identifying branches in the lineage tree.

The core of developmental biology is to understand cells’ developmental trajectories in the life of organisms and map their fate (Figure 1A). Cell fate mapping originated from the early visual tracking of cells during embryonic development, and cells are mapped to different stages of embryonic development [1]. This process is reconstructed into a lineage tree, which is a diagram of each cell ancestor back to the founding zygote. Early techniques based on observation opened up the black box of cell fate maps. However, their application was limited to transparent animals such as C. elegans. To this end, previous studies used dye injection, transplantation, viral transduction or genetic recombination to label and track cells of interest [2] (Figure 1B,C). These imaged-based methods provide a wealth of genealogical information between cells, showing how a cell develops into an individual. However, cell markers generated by these methods are difficult to maintain or cannot be resolved by light microscopy owing to the cellular density of embryos, which are difficult to trace down to the single-cell level. Advances in genomics and the development of single-cell sequencing technology have simplified cells’ identification techniques at the molecular level, which has greatly promoted the development of lineage-tracing technology [3]. The introduction of a unique cellular barcode in a single cell and the sequencing lineages identification have greatly enriched the diversity of lineage information (Figure 1C).

This review introduces traditional imaging-based methods for lineage tracing and discusses the applications and problems of several molecular markers. However, a reconstruction of the cell fate map requires rigorous lineage information to clarify the relationship between the progenitor cell, its offspring and the subsequent offspring. Thus, the review focuses on CbLT, a dynamic barcode generation system. Moreover, we review several developmental directions of the CbLT system and discuss the existing problems and development direction. Finally, we discuss the existing problems and the required technological advances.

## 2. Fate Mapping and Lineage Tracing Based on Observations and Cell Markers

**Figure 2:**
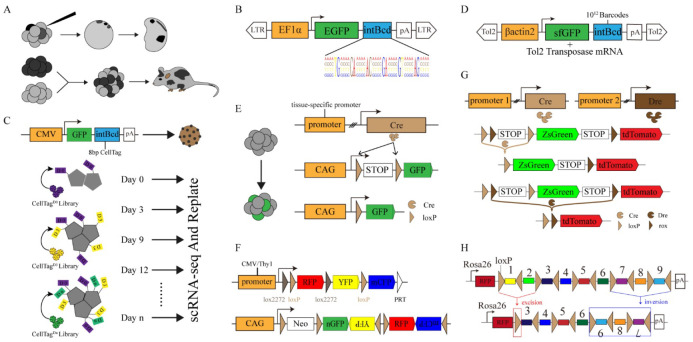
Common genetic tracing techniques. (**A**) Lineage-tracing techniques in early observational biology. Fate mapping and lineage tracing were performed through dye injection (**top**) and the generation of chimeric embryos (**bottom**). (**B**) The structure of the LARRY lentivirus consists of a DNA barcode of 28 random bases. (**C**) A schematic diagram shows the structure of CellTagging DNA barcoding (**top**), and CellTagging is used to record the dynamic information of cell lineages (**bottom**). (**D**) TracerSeq: barcode accumulation by inserting the barcode GFP reporter gene into the genome using Tol2 transposase. (**E**) Schematic diagram of Cre-*lox*P system for genetic lineage tracing. (**F**) Schematic diagram of genetic elements in multicolor reporter systems: Brainbow (**top**) and Confetti-Mouse (**bottom**). (**G**) DeaLT: Cre and Dre recombinases were expressed in different tissues to induce different fluorescent markers. (**H**) PolyloxExpress: DNA barcoding deletion or inversion is mediated by the Cre-*lox*P system.

Lineage tracing is a method to delineate all progeny produced by either a single cell or a group of cells. Lineage tracing was pioneered in the 20th century to investigate cell division (Figure 2A). Conklin developed the first comprehensive fate map of *Styela* partita by observing the fate of early cell division and differentiation [1]. Through division and differentiation, new cells are generated by the pre-existing progenitor cells, and it is a simple and accurate method to observe the dynamic fate trajectory of cells with the aid of a microscope and draw the fate mapping. However, mapping the embryo development pedigree is difficult owing to its large development period. The time-lapse microscopy development has solved this problem, and studies have used this technique to map the developmental fate of *C. elegans* and achieve accurate results [4].

The direct observation of lineage tracing is limited to early embryonic development or transparent organisms. Dyeing or radioactive labeling is required for embryos or tissues, which has been difficult to observe directly [2] (Figure 2A). To this end, Vogt developed a dye that could stain living cells and observed the fate dynamics of the cells, which mapped the fate of amphibian embryos in the early stages of development [5]. By directly observing and mapping cell fate with the help of a microscope, a highly accurate cell fate map is obtained, which includes lineage relationships and spatial context. However, some problems still limit the application of these methods, such as the limited scalability of dye injection and the difficulty of dyes remaining in cells for a long time. To address these problems, some studies used cell transplantation and embryo chimerism to achieve high scalability and long-term observation [6] (Figure 2A). The lineage relationships of many cells have been validated by observing the differentiation trajectories of embryonic cells of different species or by using different radioactive markers [7,8,9]. However, this lineage relationship is characterized by low integration and is limited by its scalability. Thus, it is difficult to construct effective lineage stratification through this technique [10].

These early image-based methods record the lineages of living organisms or tissues and retain the spatial context essential for the growth and development of organisms. However, these methods face some challenges as they can only provide genetic lineage relationships. Another problem is a lack of sufficient information to reveal the molecular mechanisms and distinguish cell heterogeneity. In this way, scanty studies explore the dynamics of cell-fate division.

## 3. Permanent Labeling through Molecular Genetics

In this study, we discuss image-based methods for mapping cell lineages through observation. However, the results obtained from these methods are less specific, and it is difficult to continuously track specific cells through this method. With the development of gene cloning and transgenic technology [11], special markers can be introduced into a few cells to observe the fate of offspring [12]. Moreover, these techniques identified progeny from a single cell and are defined as clonal analysis. However, they faced challenges in distinguishing the ancestor–descendant relationships. Therefore, they were insufficient to construct a lineage hierarchy. With the development of sequencing technology, it is possible to distinguish single-cell identity and reconstruct cell lineage trees at an unprecedented resolution (Table 1). Moreover, the introduction of expressible cellular DNA barcoding can clarify cell lineage identity and accurately analyze cell fate trajectories. Recently, an expressible DNA barcode called LARRY (Lineage and RNA recovery) has been used to study the fate determination in hematopoiesis, which is effective in revealing transcriptional differences among HSCs (hematopoietic stem cells) [13] (Figure 2B). A study used this approach to elucidate the molecular program inherent in cloning, which has been associated with functional stem cell heterogeneity. This approach can also be used to identify a mechanism for maintaining the self-renewing state of HSC [14]. However, a single introduction of barcodes cannot record the cell-fate-division process. Therefore, the method called “CellTagging” has been introduced to reconstruct the different fate trajectories of mouse embryonic fibroblasts reprogrammed to endodermal progenitor cells through multiple rounds of barcode introduction [15,16] (Figure 2C). However, CellTagging tracking efficiency is low, and it is difficult to apply this method to cell tracking in vivo. In recent years, a high-complexity expressed barcode library called watermelon has been developed to monitor the proliferation history in cancer tissue using inducible fluorescence expression and dilution [17]. In addition, TracerSeq has been used to solve the CellTagging tracking efficiency problem. TracerSeq is a method that uses Tol2 transposable enzymes to progressively insert barcodes into the genome using microinjection to record lineage data (Figure 2D). The TracerSeq showed that cells from disparate embryonic regions could produce transcriptionally similar cells; whereas cells from the same field could produce distinct cell types [18].

The discovery and application of recombinase enzymes have enhanced the rapid evolution of lineage tracing. Site-specific recombinases can induce predictable heritable variation in sequences between target sites and can be used for marking specific cell types in organisms [2]. Cre-*lox*P has been widely used to label cells. The Cre-*lox*P system works in specific cell types and connects Cre genes in tandem with cell-specific marker genes (Figure 2E). The system controls the expression of fluorescent proteins, labels specific cell populations, and tracks their offspring in vivo (Figure 1B). The system can also analyze cell proliferation, cell differentiation and cell lineages under specific physiological conditions during homeostasis or disease [38]. A single fluorescence is not sufficient to mark the heterogeneity between the population of cells. Multicolor reporter systems track individual cells by randomly inducing different fluorescent protein markers [19,20] (Figure 2F). However, it is impossible to distinguish many cell types from other cell lines using a single cell marker. Moreover, Cre is expressed in untargeted cells, which limits the systematic use of single-recombinase-mediated lineage tracing. DeaLT (dual recombinase-activated lineage tracing) technology can improve the accuracy of lineage tracing and solve this technical problem [39] (Figure 2G). A recent study has used dual-recombinase systems to reveal the proliferation and life-changing fate of cells in the livers and pancreases of adult mice [22,23]. Lineage-tracing techniques based on recombinases can distinguish between different cells at the tissue level, making the markers more stable and more visible. However, those methods lack the heterogeneity and the dynamic fate lineage relationship of different cells. In response, *PolyloxExpress*, a method using DNA barcode instead of the fluorescent protein, used Cre-*lox*P to mediate random recombination events. In association with RNA-seq, *PolyloxExpress* can capture cell identity [24,25] (Figure 2H).

In addition, cell lineage reconstruction using the spontaneous mutation of the genome has been widely used in the field of cancer research [40,41,42] (Figure 1D). In the subsequent section, we will introduce the method of lineage tracing based on mutation accumulation and discuss its existing problems and its great potential.

## 4. Using CRISPR to Map the Fate of Cells

**Figure 3:**
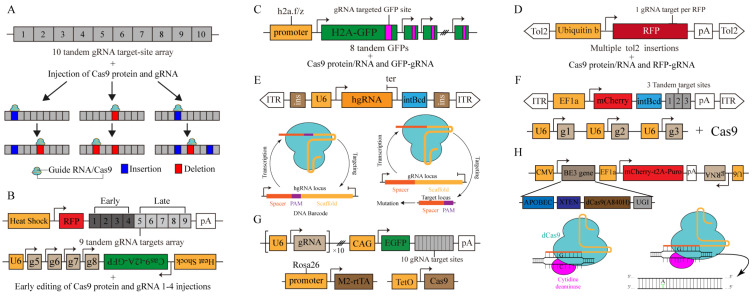
CRISPR-based dynamic barcode for single-cell lineage tracing. (**A**) GESTALT: A barcode of CRISPR/Cas9 target sites is progressively edited with cell divisions. Cas9 protein and gRNA are delivered by microinjection. (**B**) scGESTALT: CRISPR-targeted barcode site is edited over a long period. The Cas protein and gRNA 1–4 are delivered by microinjection for early editing. Post-editing and transcription of barcodes are achieved by heat shock induction at a later stage. (**C**) ScarTrace: A series of GFP sequences act as barcode editing sites and are edited progressively as cells divide. Cas9 protein and gRNA are delivered by microinjection. (**D**) LINNAEUS: The RFP reporter system serves as a barcode editing site and is randomly integrated into the genome via Tol2 transposase. (**E**) MARC1: schematic diagram of MARC1 system (**top**), principles of the homing CRISPR system (**bottom left**) and canonical CRISPR system (**bottom right**). Canonical CRISPR/Cas9 targets the locus containing a spacer and PAM, leading to mutations in the target sites. In homing CRISPR system, the hgRNA transcript is complex with Cas9 protein and leads to double-strand breaks at the hgRNA site, further resulting in dynamic barcodes due to NHEJ repair. (**F**) Chan et al.: target site (**top**) and three-guide (**bottom**) cassettes. The target site consists of an integration barcode (intBC) and three cut sites for Cas9-based recording. Cas9 protein is either injected or induced by doxycycline. (**G**) CARLIN: schematic diagram of the CARLIN system. Guide RNAs (gRNAs), target Sites and inducible Cas9 Components are integrated at specific sites in the genome. Barcode editing is induced by DOX and is captured via scRNA-seq. (**H**) Hwang et al.: base editing records lineage information. Cytidine deamination via tethering APOBEC1 and fusing with dCas targets a single chain C→U, which completes A:T base transition with cell division.

Lineage tracing based on genomic mutations can record the time track of cell division and differentiation. However, the probability of genomic mutations under natural conditions is low, which is insufficient for recording the lineage information of the cell division. Therefore, a new direction is to use gene-editing tools to generate high-frequency mutations in the genome. Advances in CRISPR have enhanced efficient and specific genome editing [43]. In addition, Cas9 protein breaks double-stranded DNA under the guidance of gRNA (guide RNA), and random mutations are introduced due to non-homologous terminal repair. Thus, artificially engineered gRNA is effective in targeting specific regions of the genome, causing mutations to accumulate as cells divide and allowing lineage tree reconstruction (Figure 1E). McKenna developed GESTALT (genome editing of synthetic target arrays for lineage tracing) to understand lineage linkages between progenitors and differentiation cells in zebrafish [26]. With this method, multiple contiguous CRISPR/Cas9 targeting arrays were synthesized to form the barcode region. Cas9 protein and gRNA were injected into the fertilized egg through microinjection. With the embryonic development, the barcode formed mutation accumulation and recorded the cytogenetic information to reconstruct the cell developmental lineage of zebrafish [26] (Figure 3A). Similarly, neutral gene mutations induced by CRISPR/Cas9 have resulted in heritable markers in *axolotl* cells, revealing the origin of limb regeneration cells [44]. However, these early techniques relied on DNA barcodes to capture lineage information, which in no way correlates cell identity with developmental information [44,45,46,47]. Additionally, the injected Cas9 protein is easily degraded in the cell, and the accumulation of barcodes is limited to the first few cleavages.

To address these issues, scGESTALT (single-cell GESTALT) combines DNA barcoding with scRNA-seq to track cell lineage relationships and cell identity (Figure 3B). The expression of Cas9 activity was induced using a promoter activated by heat shock, and long-term editing was achieved [27]. The latest version of this strategy has been applied recently to map a family tree of zebrafish and its brain development [48]. In addition, an approach called ScarTrace that targets transgenic tandem fluorescent proteins has been developed using gRNA to generate barcodes, record lineage information and distinguish the efficiency of barcode generation using fluorescence intensity [28] (Figure 3C). These methods form barcodes by concatenating multiple edit areas. Then, the method deletes large fragments, leading to a loss of record lineage information. In response, LINNAEUS (lineage tracing by the nuclease-activated editing of ubiquitous sequences) disperses target sites across the genome and solves the problem of information loss caused by the deletion of large fragments [10,29] (Figure 3D).

**Figure 4:**
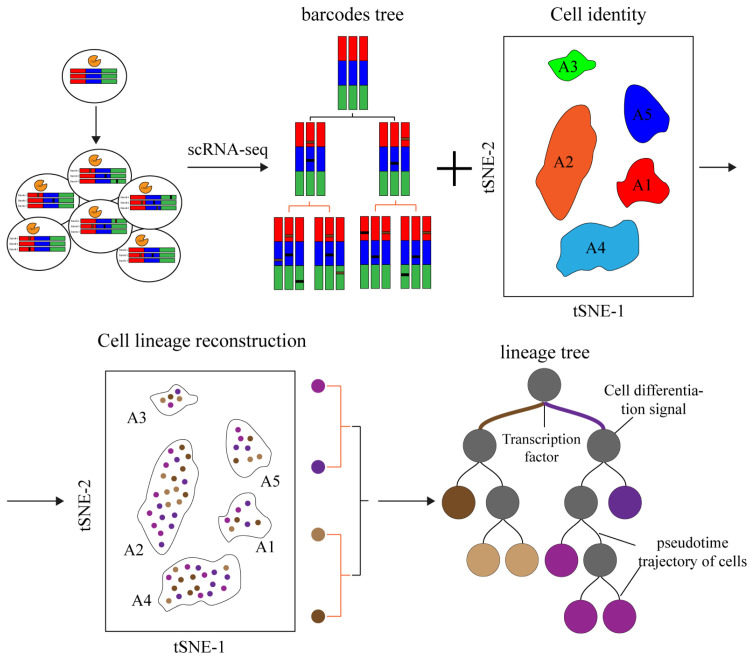
Schematic diagram of CRISPR-based lineage tracing. A stable barcode editing cassette is inserted into the cell genome, and the barcodes are cleaved by inducing Cas9 and gRNA expression through injection or transgene. Moreover, the barcodes are accumulated to mark specific cells as the cells divide. Cell identity and lineages tree are captured by single-cell sequencing, and the lineages tree during cell development is reconstructed through association analysis. Through lineage trees, we identify key transcription factors and important signaling pathways in the transformation of cell fate.

These methods provided a standardized and operatable mode for the subsequent CbLT strategy (Figure 4) and played a vital role in zebrafish research. However, many limitations are noted when applying these methods to mammals, such as mice. First, the diversity of barcode loci rapidly saturates. Second, one or a few barcode editing times do not cover a complete developmental trajectory. Third, the same barcode from different cells affects the accuracy of lineage reconstruction.

## 5. More Diverse Barcode Generation Systems for Pedigree Reconstruction

A cutting of Cas9 causes barcode editing, and it is a key factor for rapid CbLT barcode saturation, which also affects the gRNA targeting and prevents the continuous accumulation of barcode diversity. However, homing guide RNA (hgRNA), a novel CRISPR/Csa9 system, offers a solution to this problem [45,49]. This method aids Cas9-gRNA nuclease activity, directs to the gRNA site, and achieves self-cleavage of the guide RNA locus [45,49] (Figure 3E). Since the new guide RNA that has been generated after editing will continue to be transcribed, the next self-editing can occur to realize the long-term accumulation of barcodes. This method has recently been used to trace the lineage of mouse embryos during their early development. A mouse line called MACR1 (Mouse for Actively Recording Cells 1) carrying several different hgRNAs was crossed with the CRISPR/Cas9 mice line to initiate the barcode editing of zygotes and continuously record cell lineages in progeny cells [30,31]. Thus, information loss caused by large fragment knockout is avoided as hgRNA is randomly distributed throughout the genome. The barcode diversity generated by this method is sufficient to reconstruct the entire mouse developmental lineage tree [30]. However, it is impossible to correlate cell lineage information with molecular insight into the genetic program driving heterogeneous behavior because hgRNA cannot be captured by RNA-seq. Therefore, we expect to see an exciting breakthrough technology that can simultaneously read hgRNA-labeled barcodes and offer cell identity information.

CbLT provides a high degree of dynamics to reconstruct developmental lineages. However, the same editing results can lead to the same labeling of very different cells. Chan et al. combined different dynamic barcodes and add eight base-integrated barcodes upstream of variable barcodes to distinguish the editing results of barcodes [33] (Figure 3F). Their results show that endodermal cells from different origins have nearly identical transcriptomes, which supports previous observations [33,50]. Recently, He et al. developed the iTracer recorder by combining a static barcode with CRISPR-based dynamic barcoding, which was compatible with single-cell and spatial transcriptomes. Afterward, they used this method to explore the clonicity and lineage dynamics during the development of cerebral organoids, illustrating the changes in the cell fate and cell lineages during the formation of cerebral organoids [35]. The initial state of lineage information recording limits the application of CbLT in adult individuals because the microinjection or hybridization of barcode editing cannot control this initial state flexibly. Controlled barcode editing was achieved by the hybridization of mice, which induced Cas9 expression by Dox (Doxycycline) with CARLIN (CRISPR array repair lineage tracing) [32] (Figure 3G). This system was used to investigate the differences in the clonal activity of fetal liver HSCs and revealed a previously undiscovered clonal bottleneck in the response of HSC to injury. This approach can induce the barcode editing of living organisms’ lifecycles. Compared with the early CbLT, these methods improved the controllability and diversity of barcode markers and laid a foundation for the genealogy reconstruction of mammal life activities.

## 6. Limitations and Solutions of CbLT

CbLT relies on bioinformatics for data processing and experimental evaluation. Since GESTALT was developed, the dynamic CRISPR/Cas9-based barcode labeling methods have been optimized continuously. However, some problems are still observed, such as low barcode capture rate and rapid mutation. A recent study supported these limitations by using computational simulations to reveal the relationship between cell division and the frequency of CRISPR-induced mutations and assess the accuracy of lineage reconstruction [51]. These authors evaluated the performance of different CRISPR-based approaches via computer simulations and reconstructed lineage trees under different conditions [51]. Through these simulations, the authors revealed the limitations of published CRISPR-based records and proposed a series of recommendations for target numbers and mutation rates. In addition, Cassiopeia is a tailored approach for cell lineage tracing [10]. By integrating many different algorithms, an accurate genealogical reconstruction scheme is provided, which uses a simulation framework to evaluate algorithms and experimental designs. The technique is also scalable and resilient to data loss [52].

Despite computer simulation, the authors present that a slowly evolving barcode is needed to record lineage information and avoid large deletions. The recent emergence of base editors’ technologies seems to achieve this goal [53] (Figure 3H). Fusing a base editor with the dCas9 protein, while preserving the targeting capability of the CRISPR/Cas9 system, allows for the slow evolution of barcodes. The application of cytidine deaminase in cell lineages recording showed that the base editing method offers great potential [34]. A recently developed method called SMALT (Substitution mutation-Aided Lineage tracing) further improves the applicability of base editing [37]. Since base editing is accompanied by DNA replication, it is suitable for tracking cell division. However, the low efficiency of barcode generation and the capture rate limit the application of this method.

The previous paper mainly discussed the application of the CRISPR system in cell lineage reconstruction, which recorded the temporal information of cell fate regulation [10]. However, the spatial information of different cells is crucial to revealing cell migration and interaction during development. An approach called MEMOIR (mutagenesis with optical in situ readout) uses CRISPR/Cas9 to edit barcodes in cells and read and reconstruct the spatial information of cells in situ by the multiplex single-molecule RNA fluorescence hybridization technique [36]. Thus, future studies will read cell identity in situ and obtain intercellular lineage information.

## 7. Conclusions

The use of dynamic barcoding to trace single-cell lineage is unprecedented, yet this method of simultaneously capturing cell lineage and identity information during cell development could provide insights into cell fate transformation (Figure 4). Gene editing using CRISPR-Cas9 offers a new approach for generating dynamic barcodes and has broad prospects in the fields of development, regeneration and disease [54,55]. The power of the CbLT approach is that it can explicitly analyze the barcodes from individual cells without inferring and use that information to reconstruct high-confidence lineage trees. It has many limitations, however. Current CRISPR-based systems depend on transgenic alterations to integrate exogenous barcodes, the application of which is affected by the deletion or silencing of transgenic genes, resulting in low capture rates in some cases. Therefore, the development of endogenous high-expression barcoding can avoid gene silencing and, to some extent, improve the efficiency of lineage tracing [56,57]. Additionally, the scope of lineage tracing is limited by the poor efficiency of dynamic barcode sequence markers in the CRISPR system; a more efficient unique barcode-generating strategy could allow cell fate to be fully traced. Advances in sequencing, spatial transcriptome sequencing and multiomics could improve cell fate mapping if CbLT can be used in conjunction with multiomics analysis.

## Figures and Tables

**Table 1:** A summary of lineage-tracing methods barcoding techniques and their properties.

	Lineage-Tracing Technique	Resolution	DNA-Editing System	Read Out	Barcode Type	Barcode Homoplasy	In Vivo	Refs.
**Molecular Biology**	Multicolor reporter systems	Theoretically single clones	Cre–*lox*P	Microscopy	Recombination	Yes	Yes	[19,20]
	DeaLT	Theoretically single clones	Cre–*lox*P Dre-*rox*	Microscopy	Recombination	No	Yes	[21,22,23]
	Ploylox	Theoretically single clones	Cre–*lox*P	PacBio	Recombination	Yes	Yes	[24]
	PolyloxExpress	Single-cell	Cre–*lox*P	scRNA-seq; Illumina	Recombination	Yes	Yes	[25]
	LARRY	Single-cell	Retrovirus	scRNA-seq; Illumina	Integration	No	Yes	[13,14]
	TracerSeq	Single-cell	Tol	scRNA-seq; Illumina	Integration	No	Yes	[18]
	CellTagging	Single-cell	Retrovirus	scRNA-seq; Illumina	Integration	No	No	[15,16]
	Watermelon	Single-cell	Retrovirus	scRNA-seq; Microscopy	Integration	No	No	[17]
**CRISPR-Based Lineage Tracing**	GESTALT	Theoretically single clones	Cas9	Illumina	INDEL	Yes	Yes	[26]
	scGESTALT	Single-cell	Cas9	scRNA-seq; Illumina	INDEL	Yes	Yes	[27]
	ScarTrace	Single-cell	Cas9	scRNA-seq; Illumina	INDEL	Yes	Yes	[28]
	LINNAEUS	Single-cell	Cas9	scRNA-seq; Illumina	INDEL	Yes	Yes	[29]
	MACR1(hgRNA)	Theoretically single clones	Cas9	Illumina	INDEL + Integration	No	Yes	[30,31]
	CARLIN	Single-cell	Cas9	scRNA-seq; Illumina	INDEL	Yes	Yes	[32]
	Chan et al.	Single-cell	Cas9	scRNA-seq; Illumina	INDEL + Integration	No	Yes	[33]
	Hwang et al.	Single-cell	Cytidine deaminase	scRNA-seq; Illumina	C-to-T mutations	No	Yes	[34]
	iTracer	Single-cell	Cas9	scRNA-seq; Illumina	INDEL + Integration	No	No	[35]
	MEMOIR	Theoretically single clones	Cas9	FISH	INDEL	Yes	Yes	[36]
	SMALT	Theoretically single clones	Cytidine deaminase	PacBio	C-to-T mutations	No	Yes	[37]

## Data Availability

Not applicable.
